# COVID-19 and the forgone health benefits of elective operations

**DOI:** 10.1186/s12913-022-08956-6

**Published:** 2022-12-17

**Authors:** Afschin Gandjour

**Affiliations:** Frankfurt School of Finance & Management, Adickesallee 32-34, 60322 Frankfurt am Main, Germany

**Keywords:** COVID-19, Elective procedures, Intensive care unit

## Abstract

**Background and aim:**

The first SARS-CoV-2 pandemic wave in Germany involved a tradeoff between saving the lives of COVID-19 patients by providing sufficient intensive care unit (ICU) capacity and foregoing the health benefits of elective procedures. This study aims to quantify this tradeoff.

**Methods:**

The analysis is conducted at both the individual and population levels. The analysis calculates quality-adjusted life years (QALYs) to facilitate a comparison between the health gains from saving the lives of COVID-19 patients in the ICU and the health losses associated with postponing operative procedures. The QALYs gained from saving the lives of COVID-19 patients are calculated based on both the real-world ICU admissions and deaths averted from flattening the first wave. Scenario analysis was used to account for variation in input factors.

**Results:**

At the individual level, the resource-adjusted QALY gain of saving one COVID-19 life is predicted to be 3 to 15 times larger than the QALY loss of deferring one operation (the average multiplier is 9). The real-world QALY gain at the population level is estimated to fall within the range of the QALY loss due to delayed procedures. The modeled QALY gain by flattening the first wave is 3 to 31 times larger than the QALY loss due to delayed procedures (the average multiplier is 17).

**Conclusion:**

During the first wave of the pandemic, the resource-adjusted health gain from treating one COVID-19 patient in the ICU was found to be much larger than the health loss from deferring one operation. At the population level, flattening the first wave led to a much larger health gain than the health loss from delaying operative procedures.

## Introduction

An important aim of the response to the first SARS-CoV-2 pandemic wave in Germany was to postpone the wave (“flatten the curve”) to avoid overstretching intensive care capacity at the time of peak demand. To this end, additional hospital capacity was provided for the treatment of COVID-19. Specifically, the German government offered a reward to hospitals for keeping their beds empty (€560 per day and bed) and supplying additional intensive care beds (€50,000 per bed) [1]. In response to the latter incentive, ventilation machines were moved from operating rooms (ORs) to intensive care units (ICUs) to treat COVID-19 patients. In addition, personnel dedicated to elective procedures were redeployed in the ICUs. Thus, many elective operations were canceled or postponed as a consequence of these shifts but also of fears by patients of getting infected. Experts estimate that between 0.5 and 1.7 million elective surgeries were annulled in Germany over a 12-week period [2]. Given the decrease in the number of new COVID-19 cases in April and May 2020, the German government ordered the uptake of elective operations starting in May 2020 [3].

Cancellations of surgeries, together with decreases in the number of patient visits were criticized for causing greater harm than good [4]. The literature on the impact of waiting periods for elective surgeries on the quality of life of the affected patients seems to support the notion that harm can result from a delay in performing the procedures. The majority of studies published over the years appear to suggest either a stasis or a significant deterioration in the quality of life, even for waiting periods of less than 3 months [5, 6]. A number of observational studies conducted during the pandemic confirmed a loss of health or quality of life due to a postponement of elective surgery procedures (e.g., [7–9]). Cancellations were reported to have negative consequences in terms of pain, work productivity, or psychological status. Nevertheless, these studies were generally limited by a lack of control group, which would have helped to disentangle the effects of lockdown measures from the impact of surgical delays. In addition to observational studies, a few modelling studies have projected a loss of health secondary to cancellations of surgical procedures during the pandemic [10–12].

This study aimed to quantify the tradeoff between saving the lives of COVID-19 patients by providing sufficient ICU capacity and improving health-related quality of life (HRQoL) by delaying elective procedures during the first SARS-CoV-2 pandemic wave in Germany. The analysis is conducted both at the level of the individual patient and the German population and adjusts for resource use and other factors. Little quantitative analysis has been published on this important tradeoff nationally or internationally. Among the few exceptions is a simulation study that aimed at optimizing the allocation of critical care beds in view of this tradeoff [13]. A limitation of the latter study is the use of death as an endpoint given that elective surgeries primarily have an impact on HRQoL.

## Methods

I calculated quality-adjusted life years (QALYs) to facilitate the comparison between the health gains from saving the lives of COVID-19 patients and the health losses associated with postponing operative procedures. QALYs combine life-years and strength of preferences for different health states. Thus, they enable the comparison of life-extending interventions with those that improve HRQoL. The strength of preference is measured on a scale that is anchored at zero, representing death (or being dead), and 1.0, representing perfect health. A commonly used questionnaire to elicit the strength of preference is the EuroQol 5-Dimensions (EQ-5D) questionnaire [14].

I followed a five-step approach to estimate the number of QALYs lost by postponing operative procedures. First, I estimated the loss of HRQoL at the individual level as a consequence of postponing exactly one operative procedure. To this end, I assumed that an operative procedure leads to a clinically significant benefit because otherwise it would not be provided. Clinical significance is commonly operationalized as the minimal clinically important difference (MCID), which “can be defined as the smallest difference in score in the domain of interest which patients perceive as beneficial and which would mandate, in the absence of troublesome side effects and excessive cost, a change in the patient’s management” [15]. Postponing an operative procedure delays the incidence of MCID. The duration of the procedural delay is equivalent to the time required to achieve the MCID. To estimate the loss of QALYs for the delay period, the MCID was therefore multiplied by the delay period.

Second, I assumed that mortality would increase as a result of delayed surgery along with the impact on HRQoL. To this end, I defined the MCID for a survival benefit. Next, I multiplied this MCID by the 5-year overall survival rate of cancer in Germany and calculated the loss in life-years due to a clinically significant increase in mortality (assuming that the survival distribution is exponential). I focused on elective cancer surgeries, assuming that non-cancer procedures do not have a substantially different mortality impact (see also the Discussion). Furthermore, the life-years lost were adjusted for cancer-specific QoL.

Third, I accounted for a delay in procedures that represented cases of medical overuse (i.e., unnecessary care). Some elective procedures may not result in an additional benefit because the benefit does not outweigh the harm (including harm from procedural mortality), or because it is absent in the first place. When these procedures are delayed, HRQoL and survival rates do not diminish.

Fourth, I accounted for the use of hospital resources associated with operative procedures relative to those used for the treatment of COVID-19. To this end, I multiplied the QALY loss of an operative procedure by the relative resource use.

Fifth, I extrapolated the (non-resource-adjusted) QALY loss of an elective procedure to the entire population, by multiplying it by the number of canceled procedures in Germany.

For the ICU treatment of COVID-19 patients, I similarly calculated the number of QALYs gained from treating one patient in the ICU by adjusting the number of life-years gained for the associated HRQoL. Next, I extrapolated the patient-level gain to the German population. The real-world population gain from saving the lives of COVID-19 patients was brought about by admitting all patients with an indication for ICU treatment. Additionally, I considered the fact that in Germany, the first pandemic wave was successfully flattened (but not suppressed), which avoided an overstretch in ICU capacity. Thus, I modeled the QALY gain of “flattening the curve” compared to no intervention, which resulted in an uncontrolled spread of the virus and overwhelming of ICUs.

To account for uncertainty in the input data, I present two extreme-case scenarios for the QALY loss of delaying operative procedures and the QALY gain of treating COVID-19 patients.

### Data

To define the MCID for HRQoL, I used validated MCID thresholds applied by the Institute for Quality and Efficiency in Health Care (IQWiG) in Germany, which conducts early benefit assessments of new, innovative medicines. For the EQ-5D visual analogue scale (VAS), the validated MCID thresholds were 0.07 and 0.10, independent of the therapeutic area (see, e.g., [16]).

In an optimistic scenario, I presumed that the elective procedures would only be delayed by 3 months. Here, the time to achieve the MCID was likewise prolonged by 3 months. In a pessimistic scenario, I assumed that the delay would be 18 months for 50% of the procedures [17].

To obtain the MCID for a relative survival benefit, I drew on a categorization of relative added benefits published by IQWiG [18]. IQWiG classifies the extent of the added benefit as unquantifiable, minor, considerable, or major. MCID was assumed to correspond to the relative survival benefit of a minor added benefit. As the latter was not published, I used an approximation, by taking the midpoint of the published relative survival benefits for a considerable added benefit and no benefit. The MCID for a relative survival benefit was thus calculated to be 0.08 and fell between the validated boundaries for the MCIDs of the EQ-5D VAS [16]. For this comparison, the MCID of the EQ-5D VAS was interpreted as a relative measure obtained by dividing the MCID by the total range of the VAS scale. The MCID for a relative survival benefit was empirically confirmed by mortality data from a U.S. cohort study [19] that analyzed a delay in cancer treatment initiation of more than 6 weeks (based on six types of cancers and data from 2004 to 2013). It showed a 5% median absolute reduction in the 5-year overall survival rate, which implies a relative survival benefit of more than 5% in the absence of a delay. Some studies have assessed the impact of delays in cancer diagnosis (e.g., [20]); however, this needs to be distinguished from delays in cancer treatment as considered in this study.

To calculate the QALY loss from delaying cancer surgery, I obtained data on the HRQoL of cancer patients from a published literature search of cancer studies [21]. Mean EQ-5D index scores ranged from 0.33 to 0.93 and VAS scores ranged from 0.43 to 0.84 across subtypes of cancer [21]. I took the midpoint of the intervals.

Estimates of unnecessary procedures in the German health care system and internationally are notoriously difficult to obtain. A lower approximation of 10% is obtained by assuming that hospital cases have increased by 20% over the past 15 years [22], and that approximately half of the increase is potentially attributable to supply-side factors. However, it presents an underestimate because the supply-side factors may have an even stronger explanatory power for the case increase than the demand-side factors [23] and because the estimate does not take into consideration the extent of overuse that was already present 15 years ago. Assuming a 10 to 20% overuse rate 15 years ago yields a total overuse rate between 20 and 30% today. This estimate largely agrees with estimates on waste in international health-care systems, which run in the range of 20% to a third of total health-care spending [24].

To compare the hospital resource use of COVID-19 patients and the patients undergoing elective surgery, I applied the case-mix index of the German Diagnosis Related Group (DRG) system [25] (see Table 1 for input data).

**Table 1:** Input data and ranges used in the extreme-case scenarios

Input	Mean (range)	Reference
IFR of COVID-19	0.005–0.01	[26]
CMI general surgery	1.31	[25]
CMI intensive care	4.81	[25]
HRQoL of COVID-19 1 year after ICU admission	0.58	[27]
HRQoL of cancer	0.63	[21]
MCID for HRQoL benefit	0.07–0.10	[16]
MCID for relative survival benefit	0.08	Assumption
Delay in surgery (months)	3–18	[17]
5-year survival rate for all cancers	0.50	[28]
Number of canceled surgeries	493,550 – 1,702,005	[2]
Proportion of cancer surgery among all canceled surgeries	0.058	[2]
Number of ICU patients	15,395	[29]

*IFR* infection fatality rate, *CMI* case-mix index, *HRQoL* health-related quality of life, *ICU* intensive care unit, *LOS* length of stay, *MCID* minimal clinically important difference

Data on the number of canceled (elective) procedures in Germany were obtained from the CovidSurg Collaborative [2], which applied surgeons’ estimates of country-specific cancellation rates for three types of (elective) surgery (benign surgery, cancer surgery, and obstetrics) to the pre-pandemic volume of surgery by country and type of surgery. The time horizon was 12 weeks. The mean percentage reduction (73%) was largely confirmed by Bialas et al. [30], who reported a 41% reduction in all surgeries compared to before the pandemic based on a benchmarking and reporting program for surgical process data. This implies only a slightly larger absolute reduction, given that the share of elective procedures is 50%. A lower percentage reduction in elective procedures (between 50 and 80% for the selected procedures) was shown in an analysis of claims data of the largest German sickness fund [31]. On the other hand, the upper bound of the estimate (1.7 million canceled procedures) was regarded as the best estimate by Riedel [32] for the period between March 16 and May 4, 2020. According to the CovidSurg Collaborative [2], the proportion of cancer surgeries among all canceled surgeries in Germany was 5.8%, or 52,261 cases in the base case (the latter figure was confirmed by the German Cancer Aid [33]).

I took data on the average expected remaining life expectancy of a COVID-19 patient admitted to the ICU from a validated model [34]. The model was populated with age-specific mortality data from the first pandemic wave [29] set to end on July 31, 2020. According to this model, the expected gain is 1.9 life years. This estimate accounts for false-positive ICU admissions, that is, ICU overuse, and incorporates a one-year mortality of 59% in ICU survivors. The latter estimate is based on a meta-analysis of international studies on critically ill patients treated with prolonged mechanical ventilation [35]. While the one-year mortality of German ICU survivors remains to be published, the 6-month mortality of ventilated patients hospitalized in Germany between February 1 and April 30, 2020 was 52% [36] and therefore in close agreement. I took data on the number of COVID-19 ICU patients treated during the first wave [29] to obtain a population estimate on the number of QALYs gained by admitting COVID-19 patients to the ICU. To estimate QALY gains by averting ICU care by “flattening the curve” (i.e., avoiding an uncontrolled spread of the virus), I assumed an infection fatality rate (IFR) between 0.5 and 1.0% [26] and adjusted the IFR for the presence and absence of ICU care.

To the best of my knowledge, data on the utility weights of COVID-19 patients in the ICU and after their discharge were unavailable at the time of writing of this manuscript. Therefore, I used the data from a randomized controlled trial enrolling 795 patients with acute respiratory distress syndrome who were admitted to ICUs in the United Kingdom [27]. Patients’ HRQoL was measured at six and 12 months using the EQ-5D, 3-levels (EQ-5D-3L) questionnaire. In that study, EQ-5D responses were converted to utility weights using the United Kingdom value set. The results show an average HRQoL of approximately 0.58 at both time points. The one-year mortality rate was 51% and thus four percentage points below that were assumed for the model’s estimate of the survival of COVID-19 patients [34]. Therefore, the results from this study may slightly overestimate the HRQoL of COVID-19 patients, given the usual positive correlation between HRQoL and survival.

## Results

The average loss of QALYs (due to a decrease in HRQoL) from deferring one cancer or non-cancer procedure was between 0.0175 and 0.15, depending on the MCID for the EQ-5D VAS and the delay in conducting the procedures (Table 2). Accounting for an increase in mortality due to cancer procedures, procedural overuse, and lower resource consumption of surgeries compared to ICU care, the adjusted health loss increased to between 0.073 and 0.404 QALYs for one deferred operative procedure.

**Table 2:** Derivation of the number of quality-adjusted life years (QALYs) lost by delaying elective operations

Step		Lower bound	Upper bound
*Patient level*			
1	Loss of HRQoL	0.0175	0.15
2	Adjustment for loss of (quality-adjusted) life years	0.025	0.157
3	Adjustment for unnecessary care	0.020	0.110
4	Adjustment for relative resource use	0.073	0.404
*Population level*			
5	Adjustment for population size	9771	54,326

*HRQoL* health-related quality of life, *ICU* intensive care unit

A COVID-19 patient admitted to the ICU gains an average of 1.1 QALYs over the remaining lifetime (Table 3). At the individual level, the resource-adjusted QALY gain from treating one COVID-19 patient is thus predicted to be 3 to 15 times larger than the QALY loss from deferring one operation (the average multiplier is 9). At the population level, the real-world QALY gain (without resource adjustment) for treating COVID-19 patients falls within the range of the QALY loss of delaying procedures. However, the modeled QALY gain of avoiding ICU admissions by “flattening the curve” is much larger and is determined to be between 3 and 31-fold (the average multiplier is 17). Based on the estimated IFR of COVID-19, the number of deaths in the counterfactual (without “flattening the curve”) was projected to be between 289,360 and 578,719.

**Table 3:** Derivation of the number of quality-adjusted life years (QALYs) gained by treating COVID-19 patients

Step		Lower bound	Upper bound
*Patient level*			
1	Gain in life-years	1.88	
2	Adjustment for HRQoL	1.09	
*Population level*			
3	Real gain based on ICU survivors	16,784	
4	Modeled gain based on “flattening the curve”	170,121	303,946

*HRQoL* health-related quality of life, *ICU* intensive care unit

## Discussion

This study estimated that the individual QALY loss from delaying a cancer or non-cancer procedure was smaller than the QALY gain from treating a COVID-19 patient in the ICU (after adjusting for resource utilization) during the first pandemic wave in Germany. At the population level, the six digit QALY figures that need to be balanced underscore the huge tradeoffs faced during this pandemic. It is the irony of the prevention of and success in flattening the first wave that the actual population QALY gain from treating COVID-19 patients in the ICU was not larger than the population health loss from delaying the procedures. In other words, efforts to “flatten the curve” meant that fewer patients needed ICU care. For this reason, there was also less actual need to delay elective surgeries (see below). Nevertheless, the QALY gain due to averted ICU admissions from “flattening the curve” was considerably larger than the QALY loss due to the delaying of the procedures. The number of deaths in the counterfactual (without “flattening the curve”) was similar to that reported by Hsiang et al. [37] (between 370,000 and 770,000), thus supporting the validity of the projections.

Notably, the health loss from postponing procedures does not seem to be an artifact of applying the MCID threshold to estimate the decrease in HRQoL. This is because assuming a smaller loss in HRQoL would question the appropriateness of the procedures as clinical significance is not proven. The QALY gain from not delaying a surgical procedure is in line with the estimates of Rovers et al. [12] and Thorat et al. [38]. The latter study presents a systematic review of the published estimates of the QALY gain from cancer surgery. The fact that in the review, the average QALY gain of 0.3 was larger than that in this study for elective cancer surgery may be attributable to the use of different comparators, and the inclusion of emergency surgeries. The relatively small impact of cancelling elective procedures on mortality, as shown in this study, was confirmed in a real-world study conducted during the pandemic [39]. This finding also supports the assumption that the bias from excluding the mortality impact of non-cancer procedures is small.

Some of the health losses associated with delayed operative procedures may have been avoided, given that capacities in ICUs and ORs were underutilized during the first pandemic wave in Germany [40]. Based on a survey of chairs in the departments of surgery in German university hospitals [41], a part of this underutilization during the first wave was attributable to cancellations by patients, and a lack of personal protective equipment [41]. Seven percent of the chairs reported the need to postpone emergency surgeries, or transfer the emergency surgery patients [41]. However, the degree to which delays in emergency and elective surgeries were caused by a lack of personal protective equipment and other reasons related to poor disaster preparedness and management policy is unclear. Moreover, the financial incentives offered by the German government to the hospitals for keeping hospital beds empty also drove the underutilization of capacity. As stated in the Introduction, the government paid a fixed daily fee per bed to keep the ICU beds available for COVID-19 patients. Thus, the COVID-19 preparation strategy followed by the German government may have been rational at the time of decision-making; however, in the hindsight, it may be considered overly cautious. Nevertheless, some degree of unused capacity for operative procedures seems unavoidable, given the need for infection control measures to minimize the risk of exposure to SARS-CoV-2 [42]. For example, the mortality after surgery was reported to be as high as 21% among asymptomatic COVID-19 patients who were unintentionally scheduled for elective surgeries in China [43].

During the first wave, the payment for empty hospital beds did not consider the relevant cost differences between hospitals; thus, the university hospitals suffered financial losses despite the payments (while smaller and private hospitals reaped a surplus) [44]. An important lesson learned from the first wave is to maintain a staffed reserve capacity beyond the pandemic to meet potential catastrophic events [45, 46], and to determine the size of funding for surge beds based on the role of each hospital in the broader community and healthcare system.

As the analysis adjusts for resource use, it compares health gains and losses per euro spent, and thus effectively informs about the cost-effectiveness of ICU care for COVID-19 patients versus operative procedures. However, a complete assessment of cost-effectiveness would require the consideration of downstream costs after hospital discharge.

A few limitations of this study should be noted. First, it did not consider that some appointments for surgery may have been canceled without future replacements. Conversely, completing all procedures, in the long run, requires the existence of reserve capacity in hospitals; the verification of the latter would require information on the capacity utilization of ORs in Germany during the pre-pandemic period. This necessitates data on the length of surgery in minutes from the first incision to the last suture. Given this stringent requirement, only anecdotal evidence of OR utilization exists. As a second limitation, due to a lack of information, I did not consider that delaying or cancelling some procedures labeled “benign surgery” [2] may cause an increase in mortality in the long run. Examples include bariatric surgeries and hip replacement surgeries if complications of immobility such as pneumonia and deep vein thrombosis occur during the waiting period. Moreover, with regard to the “benign surgeries” [2] the potential for misclassification (i.e., false-negative risk assessments) exists. That is, these procedures may turn out to be life-saving, even in the short term. Third, despite the preoperative COVID-19 screening already being conducted at some hospitals during the first wave, there was a risk of COVID-19 infection after elective surgery, possibly requiring ICU care and decreasing the remaining ICU capacity. Incorporating these sequelae would decrease the health benefits of elective procedures and, hence, support the results of this study.

Fourth, the use of QALYs has been controversial, particularly when applied in interventions that prolong life. The “double jeopardy” argument [47] states that those with “very poor quality of life” not only suffer from their condition but also receive lower priority for life-saving treatment compared to perfectly healthy individuals. Singer et al. [48] objected to this argument in that if “the treatment can be given to only one patient, a rational egoist choosing from behind a veil of ignorance would choose to give the treatment to the patient who will gain more from it.” However, this reply was challenged by John et al. [49], who pointed out the underlying “simple utilitarian social welfare function.” Instead, John et al. [49] suggested considering not only the gain in QALYs but also wellbeing over a lifetime. Accounting for the latter would favor operative procedures given that the median age of operation in Germany is approximately 60 years ([25], p. 427), which is lower than the median age of death of a COVID-19 patient during the first wave (82 years) [29].

## Conclusion/policy recommendations

The findings of this study shed light on an important tradeoff in the coronavirus crisis and thus may contribute to the ongoing policy debates. The analysis suggests that delays in elective procedures seem justifiable when the freed-up ICU capacity allows for saving the lives of COVID-19 patients. Future analyses may also quantify the collateral damage resulting from fewer emergency and physician visits for unrelated medical conditions during the coronavirus crisis.

## Data Availability

This study is a modelling study and uses secondary data from various sources. The data sources are listed in Table 1 and included the reference list. They are publicly available.
